# Spirituality, Religiosity, and Mental Health in Patients with Idiopathic Inflammatory Myopathies: A Brazilian Multicentric Case–Control Study

**DOI:** 10.3390/ijerph21060653

**Published:** 2024-05-21

**Authors:** Jucier Gonçalves Júnior, Alexandre Moura dos Santos, Romão Augusto Alves Filgueira Sampaio, Thalita do Nascimento Silva, Giovanna Martines, Daniel Brito de Araújo, Estelita Lima Cândido, Samuel Katsuyuki Shinjo

**Affiliations:** 1 School of Medicine, Universidade Federal do Cariri, Barbalha 63180-000, Ceará, Brazil; 2 Division of Rheumatology, Faculdade de Medicina FMUSP, Universidade de São Paulo, São Paulo 01246-903, São Paulo, Brazil; 3 Division of Rheumatology, Universidade Federal do Ceará (UFC), Fortaleza 60020-181, Ceará, Brazil; 4 Division of Rheumatology, Universidade Estadual do Ceará (UECE), Hospital Geral Doutor Cesar Cals, Fortaleza 60015-152, Ceará, Brazil; 5 Division of Rheumatology, Universidade Federal de Pelotas (UFPel), Pelotas 96010-610, Rio Grande do Sul, Brazil; 6 Programa de Pós-Graduação em Ciências da Saúde, Universidade Federal do Cariri (UFCA), Barbalha 63180-000, Ceará, Brazil

**Keywords:** idiopathic inflammatory myopathies, mental health, religiosity, spirituality, systemic autoimmune myopathies

## Abstract

No published studies have investigated the correlation between religiosity, spirituality, mental health, and idiopathic inflammatory myopathy (IIM) or systemic autoimmune myopathy. Therefore, we aimed to evaluate the association between religiosity/spirituality, sociodemographic factors, and the mental health of IIM patients. This is a multicenter case–control study that included 151 patients with IIMs and 95 individuals without autoimmune diseases (controls), held between August 2022 and April 2023. This study used a semi-structured questionnaire that included sociodemographic information and the juxtaposition of the following questionnaires: the Attitudes Related to Spirituality Scale (ARES); the Duke University Religion Index (DUKE), which is composed of the organizational religious affiliation (ORA), non-organizational religious affiliation (NORA), and intrinsic religiosity (IR) domains; and the General Health Questionnaire-12 (GHQ-12). Data were analyzed using Epi Info software 7.2.5 (Centers for Disease Control and Prevention, Atlanta, GA, USA). A comparison between the mean values of the ARES, DUKE, and GHQ-12 scales was made using the Wilcoxon–Mann–Whitney and Kruskal–Wallis tests. A logistic regression test was used with the variables whose difference was statistically significant in the univariate analysis. Correlation analysis was performed using the Spearman rho coefficient. A higher prevalence of evangelicals and a lower prevalence of Catholics (*p* < 0.050) were seen in the IIM group compared to controls. Positive association was demonstrated between IIMs and the pardo ethnicity (OR = 2.26, 95% CI = 1.20–4.25, *p* = 0.011), highest ORA (OR = 2.81, 95% CI = 1.53–5.15, *p* < 0.001), NORA (OR = 3.99, 95% CI = 1.94–8·18, *p* < 0.001), IR (OR = 5.27, 95% CI = 2.32–11.97, *p* < 0.001), and ARES values (OR = 1.08, 95% CI = 1.04–1.13, *p* < 0.001). Mental health levels were compared between the groups (*p* > 0.999). Therefore, higher levels of religiosity and spirituality were observed in the IIM group than in the control group, but there was a similar distribution of mental health levels. The following can be cited as advantages of the present study: (i) the large sample for a rare disease with the presence of a control group; (ii) the multicenter characteristic with participation from three regions of Brazil; (iii) being the first study to map aspects of religiosity, spirituality, and mental health in IIMs.

## 1. Introduction

Idiopathic inflammatory myopathies (IIMs), or systemic autoimmune myopathies, are immune-mediated rheumatic diseases [1,2,3] that lead to worse quality of life and mental health [4] due to multisystem impairment in the lungs, heart, skin, muscles, and digestive tract [5].

IIMs can be classified as polymyositis (PM), dermatomyositis (DM), antisynthetase syndrome (ASSD), immune-mediated necrotizing myopathy (IMNM), and inclusion body myositis (IBM) [2,3,6,7].

PM consists of a clinical picture of progressive and symmetrical muscle weakness, predominantly of the shoulder, pelvic, and cervical girdles, with muscle biopsy demonstrating endomysial lymphomonocytic infiltrates. DM involves muscular weakness similar to PM, in addition to the presence of cutaneous involvement, such as heliotrope rash, papules/Gottron sign, V sign, holster sign, shawl sign, hyper-cuticular atrophy, and periungual telangiectasias, with muscle biopsy demonstrating lymphomonocytic infiltrates, mainly in the perivascular region, with the presence of perifascicular cell atrophy. ASSD presents objective skeletal muscle weakness, associated with persistent fever, Raynaud’s phenomenon, interstitial lung disease, “mechanic’s hands”, arthritis, and the existence of anti-tRNA synthetase autoantibodies. IMNM is characterized by acute and/or subacute weakness, with muscle biopsy showing intense necrosis of muscle fibers, with absence or rare foci of a lymphomonocytic inflammatory process. Finally, IBM causes asymmetric weakness, predominantly in the flexor muscles of the forearms and quadriceps [2,3,4,6,7,8].

Spirituality is a complex multidimensional concept that describes the way individuals seek and express meaning and purpose, and the way they experience connectedness to the moment, self, others, nature, and the significant or sacred [9,10]. Religiosity constitutes the involvement (belief, participation, and following) of an individual with a religion [11,12].

The relationship between spirituality/religiosity and chronic diseases, such as diabetes mellitus [13], chronic kidney disease [14], human immunodeficiency virus, hepatitis virus [11], neoplasms, and dementia, such as Alzheimer’s disease [15], has gained prominence over the years [16].

Over the years, the association between spirituality/religiosity and factors, such as age, sex, ethnicity, rent, and absence/presence of family support, has been studied, with conflicting results depending on the characteristics of the samples analyzed [17,18,19]. A study carried out in Greece with Orthodox Christians demonstrated that religiousness, employment, and marital status predict better quality of life and satisfaction [20]. Studies carried out in Colombia [18], Spain [21], and the US [22] demonstrated similar findings about the positive impacts of religiosity, especially on older married women.

This context appears to extend to autoimmune diseases. Moroccan and North American cohorts have shown that low income, sex [23], age [24], and uncertainty about the disease itself [25] are factors that systemically affect the mental health of patients with autoimmune diseases.

Possible effects of religiosity and spirituality were demonstrated on mental health, such as depression, anxiety, and drug substance abuse, levels of positive feelings, such as hope and optimism [14], and increased social/marital support [21]. Koening et al. [26] carried out a review of articles between 1872 and 2010, demonstrating the use of spirituality/religiosity as a coping tool for various strategies, such as improving mental health and supporting quick responses to everyday adversities, with a potentially positive impact on the treatment and well-being. These findings are corroborated by review studies published in 2015 [27] and a study carried out in Greece [28].

When evaluating the possible impacts of spirituality/religiosity on rheumatic diseases, Biccheri et al. [29] evaluated 590 French patients with fibromyalgia in a single-center, cross-sectional, and quantitative study. The authors assessed spirituality using the Evaluation de La Spiritualite and the use of coping strategies using The Ways of Coping Checklist Scale, both validated in France. In the correlation analysis, higher levels of spirituality were observed to be correlated with better measures of coping (r = 0.25, *p* < 0.001) and life satisfaction (r = 0.33, *p* < 0.001) [29].

A single-center study with 93 Portuguese patients with chronic pain for more than 50% of the time, diagnosed with common mechanical low back pain and/or osteoarthritis, assessed spirituality using the Belief into Action Scale, resilience using the Connor–Davidson Resilience Scale, and pain by the Numerical Pain Intensity Scale. The authors noticed that resilience showed a statistically significant correlation with religiosity (r = 0.21, *p* = 0.05), although weak, and a moderate correlation with positive affects (r = 0.57, *p* < 0.001) [30]. A North American study carried out at the Johns Hopkins Arthritis Center with 62 patients with rheumatoid arthritis demonstrated that spirituality assessed by the Spiritual Transcendence Scale was directly and independently correlated with positive affect (r = 0.26) and greater health perception (r = 0.29), both measured by the Affect Balance Scale [31].

The mental health of patients with IIMs has also been studied in recent years; however, the results seem conflicting. A study with 1715 patients showed no difference between IIM mental health scores, other autoimmune diseases such as rheumatoid arthritis, and control groups [1].

In parallel, a systematic review of 826 articles demonstrated worse mental health scores in patients with IIMs; however, this difference is small compared to other neuromuscular diseases, as well as the general population [4]. Notably, the collection instruments were heterogeneous, and studies on this topic are scarce [1,2,3,4,5,6,7,8,9,10,29,30,31].

While investigating the impact of spirituality/religiosity on mental health and sociodemographic variables in rheumatic diseases, only three studies have explored this topic in patients with fibromyalgia [10,29,32], a non-autoimmune disease.

Due to the severity, sequelae, and chronic state of IIMs [3,5], these patients have the potential for worse mental health. Moreover, no published articles have investigated the correlation between spirituality/religiosity and immune-mediated rheumatic diseases, as well as sociodemographic variables of mental health, particularly IIMs.

Therefore, this study aimed to evaluate the spirituality/religiosity and mental health of patients with IIMs compared to a control group as well as its association with sociodemographic variables.

## 2. Materials and Methods

### 2.1. Study Design

A multicentric cross-sectional case–control study was carried out according to the STROBE [33] protocol. The sample (not probabilistic, e.g., for convenience) consisted of patients from four outpatient clinics specializing in rheumatology at four Brazilian university hospitals in three regions of Brazil (Northeast, Southeast, and South).

The Northeast region contains nine Brazilian states in an area of 1.5 million km^2^. It contains the most varied religions—from Catholic to evangelical—with an emphasis on those of African origin (e.g., Umbanda, Candomblé) due to the Portuguese colonization that began in this region and brought enslaved people from Africa. The Southeast region contains four states and has the greatest economic development and population density (90 million inhabitants in 925,000 km^2^). Because of European, Japanese, and Latin immigrants and their influence, there is a variety of religions with Asian and Iberian influences. Finally, the Southern region is formed by three states, with an area of 576,000 km^2^. Due to its European colonization process, especially by the Germans, Dutch, Spanish, and Portuguese, Anglo-Saxon and Iberian religions are predominant there [34].

### 2.2. Eligibility Criteria

The case group consisted of subjects who (i) signed an informed consent form; (ii) were over 18 years old; and (iii) met the criteria of Connors et al. [20] for patients with ASSD, and the Bohan and Peter’ criteria [35] or/and the European League Against Rheumatism/American College of Rheumatology (EULAR/ACR) 2017 criteria [5] for patients with IIMs (DM, PM, and IMNM).

The control group consisted of individuals without rheumatic disease who (i) signed an informed consent form and (ii) were over 18 years of age.

### 2.3. Procedure

The patients were selected by rheumatologists, for convenience, at the time of the medical consultation between August 2022 and April 2023. Those who met the aforementioned inclusion criteria were asked to complete the questionnaire after the consultation.

Because IIMs are rare diseases, convenience sampling was chosen. In this case, the sample size was not calculated in this study.

Data were collected through an e-survey, prepared with the data management tool REDCap^©^, version 11.2.5 (Vanderbilt University, Nashville, TN, USA). The tool was made available and maintained by a local institution in partnership with the REDCap-Brazil Consortium.

This tool complies with all guidelines of the Brazilian General Data Protection Law (Lei de Proteção de Dados Pessoais), Law No. 13.709/2018 [36]. Finally, the data collected were kept confidential.

The e-survey can be divided into two stages. 

The first stage included socioeconomic, demographic, educational, and clinical data: current age, sex, ethnicity (white, pardo, and black), education in years (<9, 10–12, and >12), marital status (married, divorced, single, common-law marriage, and widower), rent in number of minimum wages (≤2, 3–4, 5–10, and 11–20); comorbidities (arterial hypertension, dyslipidemia, diabetes mellitus, myocardial infarction, arrhythmias, heart failure, stroke, and pulmonary disease); and subtype of IIMs.

The minimum wage at the time of data collection was BRL 1320.00 (USD 264.82).

The second stage involved the application of two questionnaires, both of which were validated in the Brazilian population:

(a) Religiosity: the Duke University Religion Index (DUREL) [37];
(b) Spirituality: the Attitudes Related to Spirituality Scale (ARES) [38];
(c) Mental health: the General Health Questionnaire-12 (GHQ-12) [39].

The three previously mentioned questionnaires were chosen for three reasons: (i) they are gold standards in screening the conditions they propose; (ii) are validated in the Brazilian population; and (iii) are easy to apply [37,38,39].

DUREL has five items that cover the three dimensions of religiosity that are most closely related to health outcomes: organizational religious affiliation (ORA), non-organizational religious affiliation (NORA), and intrinsic religiosity (IR) [37].

The first two items addressing ORA and NORA were extracted from large epidemiological studies conducted in the USA and were shown to be related to indicators of physical and mental health and social support [37]. The IR scale was best related to the total score on this scale, social support, and health outcome. In the analysis of the DUREL results, the scores in the three dimensions (ORA, NORA, and IR) must be analyzed separately, and the scores of these three dimensions must not be added to the total score [37]. On the DUREL scale, the items were scored from 1 to 6. Thus, the values of ORA (one item) and NORA (one item) ranged from 1 to 6, whereas IR (three items) ranged from 3 to 18 [37].

The ARES is a Likert scale with 11 items that are statements about aspects inherent to spirituality. The patient is invited to classify them into “totally disagree” (worth 1 point) to “totally agree” (worth five points). Thus, the score ranged from 11 points (minimum) to 55 points (maximum) [38].

The data obtained from the GHQ-12 scale were tabulated using the “GHQ scoring method”. In this approach, the first two anchors, “Better than usual” and “As usual,” scored zero, and the last two anchors, “Worse than usual” and “Much worse than usual”, scored one [39]. The first two anchors represent the absence of symptoms and, therefore, score zero, whereas the last two anchors represent the presence of symptoms and score one. All participants who scored ≥4 were considered to have mental health problems [39].

### 2.4. Data Analysis

Data were analyzed using Epi Info software (version 7.2.5, Center for Disease, Control, and Prevention, Atlanta, GA, USA), which allowed for the descriptive analysis of variables, calculation of association measures, comparison of mean scores, and hypothesis testing at a significance level of 0.05.

Subsequently, we compared the means of the IIM group and stratified them by sociodemographic characteristics, subtype of IIMs, comorbidities, and mental health-related variables (GHQ-12 scoring method).

The levels from the ARES (spirituality), DUKE (religiosity), and GHQ-12 (mental health) scales did not show a normal distribution when subjected to normality tests. Therefore, it was not possible to compare the means among the different groups using Student’s *t*-tests and analysis of variance (ANOVA), both of which were replaced by the Wilcoxon–Mann–Whitney and Kruskal–Wallis tests. Post hoc analysis was performed using Dunn’s test.

To analyze confounding biases, a multivariate analysis was performed considering sociodemographic variables, such as sex, ethnicity, education, marital status, and rent, for patients with IIMs and the control group. The contribution of each variable to religiosity/spirituality outcomes was evaluated by applying the logistic regression test for dependence and adopting the odds ratio (OR) association measure and its 95% confidence interval (CI).

Correlation analysis was performed using the Spearman rho coefficient.

The data used in the logistic regression were the variables showing a statistically significant difference between cases and controls in the univariate analysis (Table 1).

### 2.5. Ethics

The ethical criteria of the Declaration of Helsinki, the Brazilian National Health Council, and international standards were followed in the present study.

All procedures were approved by the Human Research Ethics Committee (CAAE No. 57910322.3.0000.0068).

All the patients provided written informed consent.

## 3. Results

In the present study, 151 patients had IIMs, and 95 were controls. As shown in Table 1, patients with IIMs had higher levels of spirituality (*p* < 0.001) and religiosity (ORA (*p* < 0.001), NORA (*p* < 0.001), and IR (*p* < 0.001)) when compared to the control group. It can also be observed in Table 1 that there was a predominance of black and pardo vs. white individuals in the IIM group (*p* = 0.037) with lower income (≤4 minimum wages), whereas in the control group, there were more individuals with minimum wages between 5 and 10 (*p* < 0.001).

Regarding education, the IIM group had more individuals with <12 years of formal education than the control group (*p* < 0.001). In the assessment of comorbidities, the IIM group had 90/151 individuals, with more diabetes mellitus (*p* = 0.034) and pulmonary disease observed (*p* < 0.001) (Table 1).

In the multivariate analysis, compared to the white ethnic group, the pardo ethnicity (OR = 2.26, CI 95% = 1.20–4.25, *p* = 0.011) was associated with IIMs. In relation to the control group, the highest ARES (OR = 1.08, 95% CI = 1.04–1.13, *p* < 0.001), ORA (OR = 2.81, 95% CI = 1.53–5.15, *p* < 0.001), NORA (OR = 3.99, 95% CI = 1.94–8.18, *p* < 0.001), and IR (OR = 5.27, 95% CI = 2.32–11.97, *p* = 0.001) values were associated with IIMs.

Mental health was comparable between both groups (Table 1).

In Table 2 and Table 3, the levels of religiosity in the three domains (ORA, NORA, and IR) were compared only among individuals with IIMs.

In the level of religiosity analyzed by the ORA domain, subjects with ages between 31 and 59 years (*p* = 0.023), female sex (*p* = 0.020), being married (*p* = 0.007), and having a diagnosis of diabetes mellitus (*p* = 0.013) and myocardial infarction (*p* = 0.029) had higher scores (Table 2). In the NORA domain, subjects with age between 31 and 59 years (*p* = 0.022) and female sex (*p* = 0.019) presented higher scores (Table 2), whereas in the IR domain, higher scores were observed for subjects aged 31–59 years (*p* = 0.001), being married (*p* = 0.001), and diagnosed with DM (*p* = 0.044) (Table 3).

When evaluating the types of religion among IIMs and control groups, the majority were Catholic (61/151, 40.4%), followed by evangelicals (57/151, 37.7%), spiritualists (9/151, 5.9%), witnesses of Jehovah (5/151, 3.3%), Umbanda practitioners and agnostic (4/151, 2.7% each), Candomblé practitioners, Hinduists, and atheists (1/151, 0.7% each). Eight (5.3%) subjects said they followed “other religions”.

In the control group, the majority were Catholics (53/95, 55.8%), followed by spiritualists (10/95, 10.5%), evangelicals (9/95, 9.5%), agnostics (7/95, 7.4%), atheists and Umbanda practitioners (5/91, 5.3% of each), and Candomblé practitioners and Buddhists (1/95, 1.1% of each). Four (4.2%) subjects said they followed “other religions”.

Individuals who call themselves “other religions” are deists—they believe in God or a higher being but do not identify with any formal religion.

Table 4 shows the distribution of religious orientations for IIMs and the control group. Compared to the control group, the IIM group was shown to have a higher prevalence of evangelicals (*p* < 0.001) and a lower prevalence of Catholics (*p* = 0.018), with no difference regarding those who practice spiritualism.

In the correlation analysis, higher levels of religiosity ORA (r = 0.197, *p* = 0.002), NORA (r = 0.206, *p* = 0.001), IR (r = 0.180, *p* = 0.005), and spirituality (r = 0.143, *p* = 0.024) showed a positive, although weak, correlation with the presence of comorbidities in patients with IIMs. Correlations between higher ORA levels and time since diagnosis (r = 0.170, *p* = 0.036) and NORA levels and DM (r= −0.221, *p* = 0.006) were also observed (Table 5).

## 4. Discussion

To the best of our knowledge, this is the first study to assess spirituality/religiosity, sociodemographic factors, and mental health among patients with IIMs and immune-mediated rheumatic diseases.

Patients with IIMs have higher levels of spirituality and religiosity, as well as a higher prevalence of evangelicals and a lower prevalence of Catholics compared to those in the control group, but with a similar distribution of mental health scores. ARES, ORA, NORA, and IR were independent parameters associated and correlated with IIMs. Moreover, among patients with IIMs, female sex, comorbidity (diabetes mellitus), and dermatomyositis were associated with different religiosity domains.

We can cite the advantages of the present study, such as the large sample size, considering that IIM is a rare disease; the presence of a control group; patients from three of the five Brazilian regions—Northeast, Southeast, and South; and the first study in the literature to map spirituality/religiosity in patients with IIMs stratified by sociodemographic variables and mental health.

A fact that may explain the high levels of religiosity/spirituality in the case group (IIM group) could be their older age and the higher prevalence of autoimmune myopathies in female subjects [1,2,3].

Studies have shown that the female sex and age are closely related to higher levels of religiosity/spirituality. A Spanish study carried out with 202 patients with chronic diseases, most of whom were Catholic (a sample similar to ours), showed that older age and female sex were more associated with high levels of religiosity [18]. A Colombian study with 18,871 people also found higher levels of religiosity among older and married women [21]. Brazilian [17,19] data also show the positive influence of age, marital status, and gender on spirituality.

Similar findings can be found in works carried out in Greece [20,28], France [29], Portugal [30], and Germany [31]. In fact, some authors point to a positive association between higher levels of religiosity/spirituality and happiness [28,40], which, perhaps, is a new frontier of knowledge to be explored in patients with autoimmune diseases

The three most prevalent religions in our sample, Christianity, evangelism, and spiritualism, have in common that they are religions that follow a single God. However, they differ regarding precepts: (1) resurrection; (2) reincarnation; (3) the possibility of the existence of saints, as well as mysteries such as that of the Virgin Mary [34].

Concerning the influence of diabetes mellitus, dermatomyositis, and polymyositis on higher religiosity scores, we believe that the high mortality and morbidity of IIMs, as well as their multisystem impairment [1], are associated with the use of chronic glucocorticoids to develop diabetes mellitus and its complications [3,5]. The sequelae and chronic use of glucocorticoids in IIMs prompt the search for coping strategies, and religiosity is one of them [13]. This reasoning may also explain the association between longer IIM times at diagnosis and higher levels of organizational religious affiliation in our sample.

In addition, previous studies have demonstrated a tendency for higher levels of religiosity in patients with chronic diseases as a method of coping. Mishra et al. [41] state that beliefs can be associated with better quality of life and combating chronic diseases. In our sample, there was a positive correlation between the presence of comorbidities and higher levels of religiosity/spirituality.

As for the influence of ethnicity on higher levels of spirituality/religiosity, recent studies have demonstrated that ethnic minorities with low socioeconomic levels, such as Latinos, have higher levels of spirituality [14,17,18,19,21,42]. Furthermore, Dy-Liacco et al. [43] reinforce the nuances and difficulties of assessing religiosity/spirituality in different peoples due to cultural, social, and political variations. In this context, studies carried out in Israel, Germany [10] Pakistan, and the US [11], with patients with the Hepatitis C virus [11,29] and fibromyalgia [10,32] did not observe a significant influence of religiosity on the health of these populations. These findings are consistent with ours, in which patients with IIMs showed a greater association with the pardo ethnicity in the multivariate analysis.

The literature shows that the mental health of patients with IIMs appears to be impaired. Goreshi et al. [44] associated poorer mental health scores in 120 patients with dermatomyositis and greater skin involvement. Feldon et al. [1] showed poorer mental health scores in patients with IIMs compared to patients with rheumatoid arthritis. A Canadian study showed poorer mental health scores in patients with IIMs compared to patients with systemic sclerosis and rheumatoid arthritis [45]. However, these findings are not a consensus [1,4].

In our sample, mental health levels were comparable between the groups. We believe that two factors may be associated with this finding: (i) the small sample size of the case group and (ii) the worse mental health of the Brazilian population.

According to the Brazilian government, the number of people over 18 years old diagnosed with depression increased by 34% between 2013 and 2019, reaching 16.3 million people in 2019. A proportional increase of 75% was seen among 15–19 year olds, with a success rate of 22% for men and 28% for women, compared to 2011–2019 [46]. In addition, in the new World Health Organization report, Brazil led the ranking of anxious people in 2022 [47]. These factors are supported by the socio-economic situation in Brazil, with high rates of urban violence, food insecurity, lack of access to healthcare services, loss of purchasing power/wage devaluation, and unsafe urban planning [48].

## 5. Limitations

Finally, as limitations of the present study, we can mention the following: (i) mapping qualitative aspects through quantitative analysis; (ii) the inability to formulate a concrete causal link due to the limitations inherent to its design; (iii) regional socioeconomic, political, and cultural variations in Brazil, which must be considered and stratified in future studies, as they can impact the spirituality/religiosity of individuals; (v) failure to map disease activity using instruments validated in the literature; and (vi) inability to formulate generalizations due to working with a non-probabilistic sample.

## 6. Conclusions

Brazilian patients with IIMs showed higher levels of religiosity and spirituality compared to the control group. Mental health levels were comparable between the groups.

Higher religiosity scores were observed in dermatomyositis, female subjects with lung disease, and diabetes.

However, it is important to understand the non-probabilistic characteristic of the sample (because it is a rare disease), as well as the cultural and social differences present in the Brazilian territory in other countries and the absence of other IIM studies with which we can compare our results. This, in itself, makes generalization difficult.

Therefore, other longitudinal studies with larger sample sizes stratified by cultural variables in Brazil and other countries are necessary to evaluate the possibility of generalizing the data.

## Figures and Tables

**Table 1:** General characteristics of idiopathic patients with idiopathic inflammatory myopathy and the control group.

Parameters	IIM (*n* = 151)	Controls (*n* = 95)	*p*-Value
Demographic data			
Age ± SD (years)	48.0 ± 12.1	45.7 ± 14.1	0.174
Female	115 (76.2)	72 (75.8)	>0.999
Male	36 (23.8)	23 (24.2)	>0.999
Ethnicity			
White	71 (47.0)	63 (66.3)	0.037
Pardo	31 (20.5)	7 (7.4)	
Black	49 (32.5)	25 (26.3)	
Rent (number of minimum wages)			
≤2	72 (47.7)	19 (20.0)	<0.001
3–4	50 (33.1)	22 (23.2)	
5–10	24 (15.9)	28 (29.5)	
11–20	5 (3.3)	26 (27.3)	
Education (years)			
<9	36 (23.8)	10 (10.5)	<0.001
10—12	73 (48.4)	21 (22.1)	
>12	42 (27.8)	64 (67.4)	
Marital status			
Married	77 (51.0)	40 (42.1)	0.191
Divorced	23 (15.2)	6 (6.3)	
Single	39 (25.8)	33 (34.7)	
Common-law marriage	7 (4.6)	14 (14.7)	
Widower	5 (3.3)	7 (2.1)	
Comorbidities			
Arterial hypertension	55 (36.4)	19 (20.0)	0.070
Dyslipidemia	37 (36.4)	17 (17.9)	0.269
Diabetes mellitus	22 (14.6)	5 (5.3)	0.034
Myocardial infarction	22 (14.6)	1 (1.1)	>0.999
Arrhythmia	8 (5.3)	3 (3.2)	0.537
Heart failure	1 (0.7)	1 (1.1)	>0.999
Stroke	1 (0.7)	1 (1.1)	>0.999
Pulmonary disease	29 (19.2)	2 (2.1)	<0.001
Mental health problem (score ≥ 4)	133 (88.1)	83 (87.4)	>0.999
ARES score (range)	53 (50–55)	50 (43–55)	<0.001
DUREL scale			
ORA	95 (74.2)	33 (25.8)	<0.001
NORA	132 (69.1)	59 (30.9)	<0.001
IR	139 (68.1)	65 (31.9)	<0.001

Data are expressed as mean ± standard deviation or frequency (%). Keys: ARES: the Attitudes Related to Spirituality Scale; DUREL: the Duke University Religion Index; IIM: idiopathic inflammatory myopathy; IR: intrinsic religiosity; NORA: non-organizational religious affiliation; ORA: organizational religious affiliation.

**Table 2:** Analysis of organizational religious affiliation (ORA) and non-organizational religious affiliation (NORA) among the group of patients with idiopathic inflammatory myopathies.

Parameters	ORA	*p*-Value	NORA	*p*-Value
Demographic data				
Age				
18–30	3.2 ± 1.5	0.023	4.1 ± 1.8	0.022
31–59	4.3 ± 1.6		4.9 ± 1.1	
≥60	3.8 ± 1.9		4.6 ± 1.5	
Female	4.2 ± 1.5	0.020	4.9 ± 1.1	0.019
Male	3.2 ± 1.9		4.3 ± 1.5	
Ethnicity				
White	3.9 ± 1.7	0.489	4.6 ± 1.4	0.215
Pardo	4.2 ± 1.7		4.8 ± 1.3	
Black	4.3 ± 1.6		5.2 ± 0.9	
Rent (number of minimum wages)				
≤2	4.1 ± 1.8	0.329	4.8 ± 1.3	0.610
3–4	4.2 ± 1.5		5.0 ± 1.1	
5–10	3.6 ± 1.8		4.5 ± 1.6	
11–20	5.0 ± 1.4		5.0 ± 1.0	
Education (years)				
<9	3.7 ± 1.6	0.369	4.9 ± 1.3	0.534
10—12	4.1 ± 1.6		4.9 ± 1.0	
>12	4.0 ± 1.6		4.6 ± 1.7	
Marital status				
Married	4.3 ± 1.7	0.007	4.8 ± 1.3	0.147
Divorced	4.3 ± 1.6		5.0 ± 0.7	
Single	3.7 ± 1.6		4.6 ± 1.4	
Common-law marriage	2.1 ± 1.6		4.0 ± 1.7	
Widower	5.0 ± 1.2		5.8 ± 0.4	
Type of idiopathic inflammatory myopathies				
Dermatomyositis	3.8 ± 1.8		4.5 ± 1.5	
Polymyositis	4.3 ± 1.6		5.1 ± 0.7	
Immune-mediated necrotizing myopathy	4.4 ± 1.5	0.435	5.0 ± 1.4	0.179
Antisynthetase syndrome	4.3 ± 1.6		5.2 ± 0.8	
Comorbidities				
Arterial hypertension				
Yes	4.3 ± 1.5	0.111	5.0 ± 1.0	0.052
No	3.9 ± 1.7		4.6 ± 1.3	
Dyslipidemia				
Yes	4.1 ± 1.7	0.860	4.8 ± 1.2	0.733
No	4.1 ± 1.6		4.7 ± 1.3	
Diabetes mellitus				
Yes	4.9 ± 1.5	0.013	5.0 ± 0.9	0.342
No	3.9 ± 1.6		4.7 ± 1.3	
Myocardial infarction				
Yes	2.0 ± 1.7	0.029	3.6 ± 2.3	0.127
No	4.1 ± 1.6		4.8 ± 1.2	
Arrhythmia				
Yes	3.4 ± 1.6	0.217	5.2 ± 0.4	0.317
No	4.1 ± 1.7		4.7 ± 1.3	
Heart failure *				
Yes	5.0 ± 0.0	-	6.0 ± 0·0	-
No	4.0 ± 1.6		4.7 ± 1.3	
Stroke *		-		
Yes	3.0 ± 0.0	-	5.0 ± 0·0	-
No	4.1 ± 1.6		4.8 ± 1.3	
Pulmonary disease				
Yes	4.4 ± 1.6	0.207	5.1 ± 1.2	0.219
No	4.0 ± 1.6		4.7 ± 1.3	
Mental health problem (score ≥ 4)	4.0 ± 1.7	0.206	13.4 ± 2.5	0.108
No Mental Health problem (score < 4)	4.5 ± 1.8	0.276	14.1 ± 2.5	0.249

Data are expressed as mean ± standard deviation. * The *p*-value could not be calculated, as there was only one individual in the group with the disease.

**Table 3:** Analysis of intrinsic religiosity (IR) among the group of patients with idiopathic inflammatory myopathies.

Parameters	IR	*p*-Value
Demographic data		
Age (years)		
18–30	11.7 ± 3.3	0.001
31–59	13.9 ± 2.1	
≥60	13.2 ± 2.6	
Female	13.6 ± 2.09	0.155
Male	13.0 ± 3.3	
Ethnicity		
White	13.5 ± 2.5	0.800
Pardo	13.6 ± 2.3	
Black	13.4 ± 2.8	
Rent (number of minimum wages)		
≤2	13.6 ± 2.4	0.475
3–4	13.8 ± 1.8	
5–10	12.8 ± 3.5	
11–20	13.2 ± 2.5	
Education (years)		
<9	14.1 ± 2.7	0.642
10—12	13.6 ± 1.9	
>12	13.4 ± 2.5	
Marital status		
Married	13.7 ± 2.5	0.001
Divorced	14.2 ± 1.7	
Single	13.4 ± 2.0	
Common-law marriage	10.0 ± 4.5	
Widower	14.6 ± 0.9	
Type of idiopathic inflammatory myopathies		
Dermatomyositis	13.3 ± 2.9	
Polymyositis	14.5 ± 1.0	0.044
Immune-mediated necrotizing myopathy	13.2 ± 2.4	
Antisynthetase syndrome	13.5 ± 2.2	
Comorbidities		
Arterial hypertension		
Yes	13.8 ± 2.0	0.204
No	13.4 ± 2.6	
Dyslipidemia		
Yes	13.8 ± 2.0	0.436
No	13.4 ± 2.5	
Diabetes mellitus		
Yes	13.5 ± 2.5	0.940
No	13.5 ± 2.4	
Myocardial infarction		
Yes	12.3 ± 4.6	0.393
No	13.5 ± 2.4	
Arrhythmia		
Yes	12.7 ± 2.6	0.354
No	13.5 ± 2.4	
Heart failure *		
Yes	15.0 ± 0.0	-
No	13.5 ± 2.4	
Stroke *		
Yes	14.0 ± 0.0	-
No	13.5 ± 2.4	
Pulmonary disease *		
Yes	14.0 ± 1.8	0.226
No	13.4 ± 2.5	
Mental health problem (score ≥ 4)	13.5 ± 2.5	0.134
No mental health problem (score < 4)	14.1 ± 1.7	

Data are expressed as mean ± standard deviation. * The *p*-value could not be calculated, as there was only one individual in the group with the disease.

**Table 4:** Analysis of the religious orientation among the idiopathic inflammatory myopathies and control groups.

Religious Orientation	IIM*n* (%)	Control*n* (%)	*p*-Value
Catholic	61 (40.4)	53 (55.7)	0.018
Evangelicals	57 (37.8)	9 (9.5)	<0.001
Spiritualists	9 (5.9)	10 (10.5)	0.192

Data are expressed as frequency (%).

**Table 5:** Correlation analysis.

	ARES *	ORA *	NORA *	IR *
Comorbidities	0.143	0.197	0.206	0.180
*p*-value	0.024	0.002	0.001	0.005
Subtypes of idiopathic inflammatory myopathies				
Polymyositis	0.138	0.059	0.064	0.141
*p*-value	0.090	0.469	0.438	0.085
Dermatomyositis	0.016	−0.144	−0.221	−0.026
*p*-value	0.844	0.077	0.006	0.755
Antisynthetase syndrome	−0.079	0.067	0.116	−0.027
*p*-value	0.333	0.414	0.155	0.741
immune-mediated necrotizing myopathy	−0.073	0.064	0.110	−0.077
*p*-value	0.374	0.437	0.180	0.349
Diagnosis time (years)	−0.002	0.170	0.051	0.057
*p*-value	0.979	0.036	0.538	0.485
Treatment				
Glucocorticoid use	0.006	0.001	−0.036	0.035
*p*-value	0.938	0.988	0.665	0.667
Use > 1 immunosuppressant	0.017	−0.056	0.005	−0.070
*p*-value	0.844	0.530	0.954	0.429

Legend: ARES: Attitudes Related to Spirituality Scale; DUREL: Duke University Religion Index; IR: intrinsic religiosity; NORA: non-organizational religious affiliation; ORA: organizational religious affiliation. * Spearman’s Rho.

## Data Availability

The data originating from this research is part of a larger database, whose data has not yet been published in its entirety. The authors make themselves available to fellow researchers to share data on a case-by-case basis.
